# Local Intra-arterial Vasodilator Infusion in Non-Occlusive Mesenteric Ischemia Significantly Increases Survival Rate

**DOI:** 10.1007/s00270-020-02515-4

**Published:** 2020-05-22

**Authors:** Robert Winzer, Dieter Fedders, Moritz Backes, Till Ittermann, Matthias Gründling, Birger Mensel, Hanns-Christoph Held, Marie-Luise Kromrey, Jürgen Weitz, Ralf-Thorsten Hoffmann, Robin Bülow, Jens-Peter Kühn

**Affiliations:** 1 Institute and Policlinic for Diagnostic and Interventional Radiology, University Hospital, Carl Gustav Carus University, TU Dresden, Fetscherstr. 74, 01307 Dresden, Germany; 2 Institute for Community Medicine, University Medicine Greifswald, Domstraße 11, 17489 Greifswald, Germany; 3 Department of Anesthesia and Intensive Care, University Medicine Greifswald, Ferdinand-Sauerbruch-Straße, 17475 Greifswald, Germany; 4 Zentrum für Diagnostische/Interventionelle Radiologie Und Neuroradiologie, Zentralklinik Bad Berka, Robert-Koch-Allee 9, 99438 Bad Berka, Germany; 5 Visceral, Thoracic and Vascular Surgery, Carl Gustav Carus University, TU Dresden, Fetscherstr. 74, 01307 Dresden, Germany; 6 Institute of Diagnostic Radiology and Neuroradiology, University Medicine Greifswald, Ferdinand-Sauerbruch-Straße, 17475 Greifswald, Germany

**Keywords:** Mesenteric ischemia, Non-occlusive mesenteric ischemia, Papaverine, Infusion therapy, Patient outcome assessment

## Abstract

**Purpose:**

To investigate the outcome of local intra-arterial papaverine infusion therapy in patients with non-occlusive mesenteric ischemia (NOMI), and factors influencing survival, in comparison with a conservative approach.

**Methods:**

From 2013 to 2019*,* patients with NOMI confirmed by imaging were included in a retrospective two-center study. According to different in-house standard procedures, patients were treated in each center either conservatively or interventionally by a standardized local infusion of intra-arterial papaverine into the splanchnic arteries. Thirty-day mortality and factors influencing the outcome, such as different demographics and laboratories, were compared between groups using Kaplan–Meier survival analysis and Cox regression, respectively.

**Results:**

A total of 66 patients with NOMI were included, with *n* = 35 treated interventionally (21 males, mean age 67.7 ± 12.3 years) and *n* = 31 treated conservatively (18 females, mean age 71.6 ± 9.6 years). There was a significant difference in 30-day mortality between the interventional (65.7%; 12/35 survived) and the conservative group (96.8%; 1/31 survived) (hazard ratio 2.44; *P* = 0.005). Thresholds associated with a worse outcome of interventional therapy are > 7.68 mmol/l for lactate, < 7.31 for pH and <  − 4.55 for base excess.

**Conclusion:**

Local intra-arterial papaverine infusion therapy in patients with NOMI significantly increases survival rate in comparison with conservative treatment. High lactate levels, low pH and high base excess, and high demand for catecholamines are associated with a poor outcome.

**Level of Evidence:**

Level III.

## Introduction

Despite the progress in intensive care medicine, acute mesenteric ischemia is still a potentially lethal event due to insufficient blood supply of intestinal tissue over a critical time period [1]. Mesenteric ischemia can either result from arterial occlusion or be non-occlusive and result from severe lasting vasoconstriction (NOMI). For the latter form, a profound drop of systemic blood pressure is seen as causative for reflexive mesenteric arterial vasoconstriction and consecutive ischemia [2, 3].

A mismatch of supply and demand in the intestine as a result of persistent mesenteric vasoconstriction leads to reduced blood flow as well as oxygen delivery particularly to the vulnerable superficial mucosa [4].

Therapy of shock in intensive care requires, among other things, the administration of catecholamines for circulatory stabilization. The aforementioned mismatch is amplified by the vasoconstrictive effect of catecholamines, starting a vicious circle. Left untreated, this can lead to gangrene of the intestinal wall, sepsis and multiple organ failure within a short time. Therefore, early diagnosis is crucial.

Generally, NOMI is diagnosed by computed tomography (CT) and confirmed by selective catheter angiography [5] with a further potential treatment option. Therefore, a catheter is placed in the affected vessel and a vasodilator is continuously infused over hours, resulting in vasodilation of splanchnic arteries*.*

At this time, interventional therapy is recommended by several boards [5]. These recommendations are based on few case reports with only a very limited number of patients [6–8]. Besides, these studies differ even concerning the type of vasodilator and the dosage used [9]; no comparative studies on different treatment protocols have been reported. Furthermore, there are no comparative studies about the outcome for patient groups which received interventional, versus noninterventional, treatment.

Therefore, the purpose of this study was to assess the outcome of local intra-arterial papaverine infusion therapy as an acute treatment in patients with NOMI, and factors influencing survival, in comparison with a conservative approach.

## Material and Methods

The two-centered retrospective study was approved by both institutional review boards. The radiology information system of two university hospitals was searched for patients requiring intensive care treatment with an increasing demand for catecholamines, who had been diagnosed with NOMI by computed tomography from January 2013 to 2019, as shown in Table 1.

**Table 1:** Inclusion and exclusion criteria

Inclusion criteria	Exclusion criteria
Intensive care treatment with an increasing demand for catecholamines	SMA occlusion at angiographic CT phase
Clinical/laboratory indications for bowel ischemia	SMV occlusion at portal venous CT phase
Signs of hypoperfusion and elevation of serum lactic acid without evidence of any other diagnosis that could explain these findings	Bowel obstruction at CT
Biphasic abdominal CT	
Age > 18 years	
SMA, superior mesenteric artery; SMV, superior mesenteric vein. CT with both angiographic and portal venous phases	

Due to different in-house standard operation procedures (SOP) for treatment for NOMI, patients received a “conservative therapy” at one center and additional local intra-arterial vasodilator infusion at the other center, described below as “interventional therapy.”

### Intra-arterial Infusion of Vasodilators

In general, mesenteric angiography was performed via the femoral artery using a 2.7F, 4F or 5F introducer. Visceral arteries were intubated using a Cobra catheter (C2, Cordis, Baar, Switzerland) or SIM catheter (SIM1, Cordis, Baar, Switzerland).

NOMI was diagnosed if at least one of the following pathological findings was evident [9]:

- narrowing of the origins of branches of the superior mesenteric artery;
- irregularities in the intestinal branches;
- spasm of the arcades; and/or
- impaired filling of intramural vessels.

In cases of angiographic evidence of NOMI, the inserted diagnostic catheter was directly used for intra-arterial infusion of papaverine hydrochloride into the affected artery, i.e., the superior mesenteric artery or proper hepatic artery, respectively. If a stable position of the catheter was not possible, a microcatheter (Progreat 2.7 French, Terumo Cooperation, Tokyo, Japan) was placed in the affected vessel. After catheter placement, the vasodilator papaverine hydrochloride (Paveron N, 50 mg/2 ml, Linden Arzneimittel-Vertrieb-GmbH, Rastatt, Germany) was administered according to a predefined scheme: 50 mg of Paveron N was dissolved in 50 ml of isotonic saline applied for 1 h. Subsequently, another 150 mg of Paveron N in 50 ml of isotonic saline was infused over 6 h. If the patient's clinical symptoms did not improve, the second part of the scheme (150 mg of Paveron N in 50 ml of isotonic saline) was repeated. The duration of papaverine infusion was based on the individual course and continued until clinical improvement or death. Clinical improvement was determined by the primarily treating intensive care specialist. Criteria of clinical improvement were hemodynamic recovery (norepinephrine dose reduction), a decrease in organ dysfunction (SOFA score) and resolution of paralytic ileus (bowel movement). In general, no additional anticoagulation was provided unless required by preexisting diseases.

### Diagnostics and Therapy Approach at the Conservative Center

**Fig. 1:**
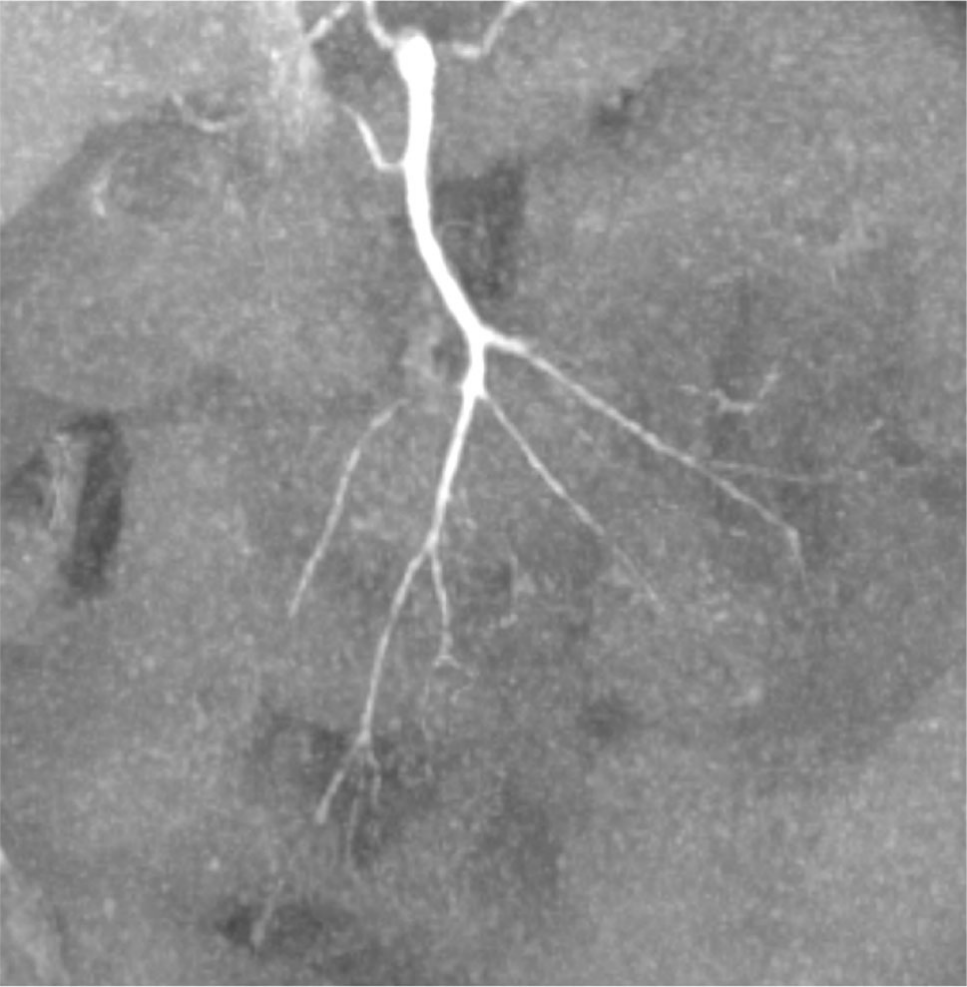
Coronary maximum intensity projection (MIP) of a patient with mesenteric ischemia confirmed by surgery: irregular stenosis of SMA branches and poor visualization of intestinal arcade. SMA—superior mesenteric artery

At the conservative center, diagnosis of NOMI was based on clinical findings and by interpreting morphologic appearance and diameter of SMA [10], in conjunction with previous CT scans, where available, [11] as shown in Fig. 1.

The conservative therapeutic approach is primarily based on rapid diagnosis and fluid therapy before a systemic inflammatory response can develop. Fluid therapy aims to restore adequate organ and tissue perfusion [12, 13].

### Clinical Parameters

Demographics, such as age and gender, laboratory parameters (e.g., lactate serum level, pH, base excess), SOFA score and comorbidities were documented on the day of diagnosis in order to determine a possible influence on survival. If the laboratory parameters showed a significant influence on survival, these parameters are described in the Results section.

Catecholamine noradrenaline dose was evaluated directly before treatment and within 24 h after treatment or in the follow-up of conservative therapy, respectively.

Finally, we recorded the following comorbidities: tumor, cardiovascular, metabolic, pulmonary, renal and other diseases.

### Statistical Analysis

Statistical analysis was performed using Stata 15.1 (Stata Corporation, College Station, TX, USA).

Characteristics of the study population are given by means and standard deviations for continuous variables and as absolute numbers for categorical variables stratified by group.

Thirty-day mortality was the primary endpoint of this study. Differences in mortality between interventional and conservative groups were analyzed by Kaplan–Meier survival analysis, log-rank testing and Cox regression. If there was significant group difference, Kaplan–Meier analysis was adjusted for the differing covariate [14]. In the interventional group, we analyzed associations of laboratory biomarker concentrations before intervention, including lactate, pH and base excess with 30-day mortality by Cox regression. For these biomarkers, we calculated optimal cutoffs discriminating best for 30-day mortality by maximizing the Youden index (sensitivity + specificity—1). In all analyses, a *p* < 0.05 was considered as statistically significant.

## Results

Thirty-five patients (21 male, average age of 67.7 ± 12.3 years) of the interventional therapy group matched the inclusion criteria with diagnostic findings compatible with NOMI.

Interventions were performed by five interventional radiologists with 1–12 years of experience. The average duration (table time) of the diagnostic angiography and the time for catheter placement for papaverine hydrochloride treatment were 43 ± 27 min (range: 15–135 min). Only one patient had a peri-interventional dissection of the external iliac artery caused by chronic stenosis, which was successfully treated with a stent.

**Fig. 2:**
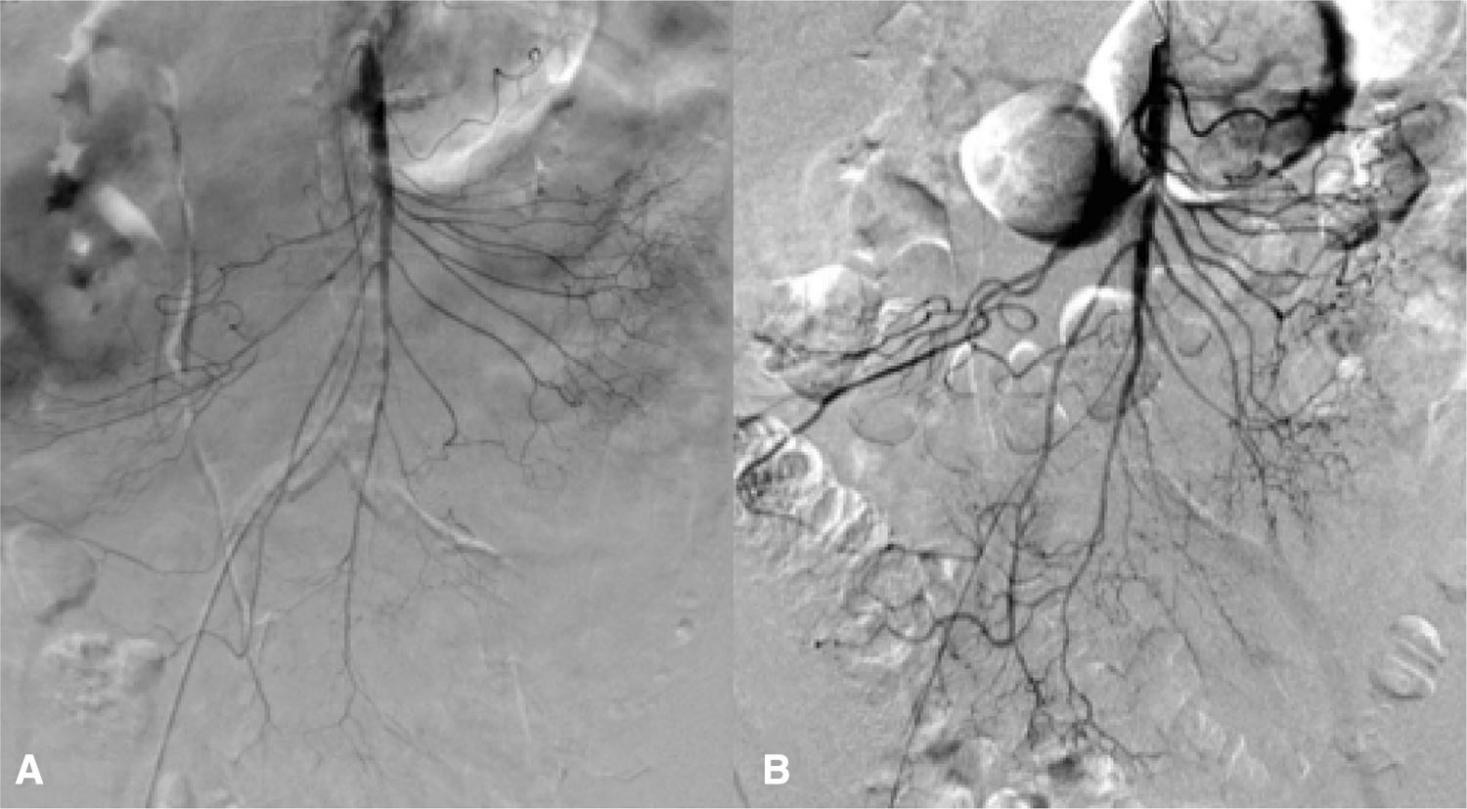
Angiography of the superior mesenteric artery in a patient with NOMI: Irregularities in the intestinal branches and spasm of the arcades confirmed the diagnosis of NOMI (**A**). There were no more abnormalities of vascular branches visible 6 h after local infusion of papaverine (**B**)

In the majority of patients, the catheter was placed in the mesenteric artery (32/35 patients). In three cases, the catheter was positioned into the hepatic artery, because vasoconstriction was most pronounced there. Interventional radiologists used the following catheter for intra-arterial infusion: 2.7F microcatheter (*n* = 16/35; 45.7%); 4F diagnostic catheter (*n* = 14/35; 40.0%); and 5F diagnostic catheter (*n* = 5/35; 14.3%). Patients had a mean papaverine hydrochloride infusion time of 26 ± 26 h (range: 1–101 h). An example of successful interventional treatment is shown in Fig. 2.

In the conservative study center, 31 patients (18 male, average age of 71.6 ± 9.6 years) matched the inclusion criteria with diagnostic findings compatible with NOMI.

**Fig. 3:**
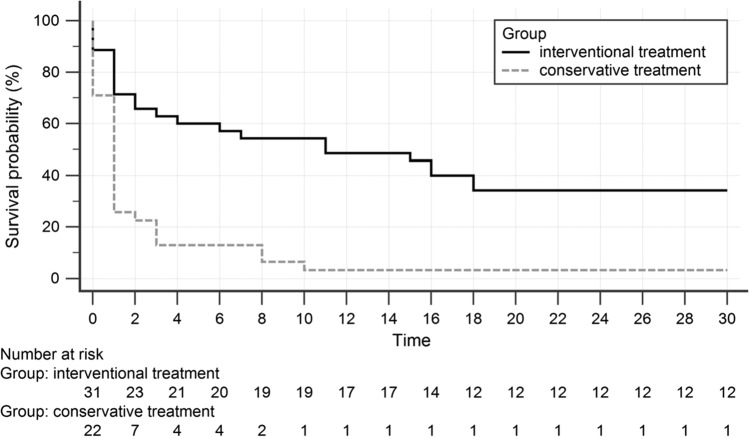
Kaplan–Meier plot for 30-day mortality shows significant differences between patients with NOMI treated interventionally and conservatively (*p* = 0.005)

A comparison of both groups revealed only a significant difference for lactate (interventional group: 8.8 ± 6.8 mmol/l, conservative group: 12.7 ± 7.9 mmol/l; *P* = 0.025). After adjusting the survival analysis for the covariate lactate, there was still a significant survival benefit for patients treated with intra-arterial papaverine (*p* < 0.05). Figure 3 presents the Kaplan–Meier plot for 30-day mortality.

For the interventional treatment group, post-interventional 30-day mortality was 65.7% (12 surviving patients), while patients with conservative therapy had a 96.8% mortality (only one surviving patient), revealing a significant difference (hazard ratio 2.44; *P* = 0.005, adjusted for the covariate lactate).

For all other demographics, laboratory data, the SOFA score, catecholamine doses and comorbidities, no significant differences were found, as shown in Table 2.

**Table 2:** Demographics and clinical parameters of patients with NOMI in interventional and conservative treatment

	Therapy		
	Interventional (mean ± standard deviation)	Conservative (mean ± standard deviation)	*p*
*n*	35	31	
Age in years	67.7 ± 12.3	71.6 ± 9.5	0.225
Gender (f/m)	14/21	18/13	0.143
SOFA score	10.94 ± 2.5 (two missing)	10.93 ± 2.9 (one missing)	0.993
Lactate in mmol/l	8.8 ± 6.8	12.7 ± 7.9	0.025
Noradrenaline dose in mg/kg/body weight	0.0019 ± 0.0025	0.0012 ± 0.0014	0.667
Base excess	− 4.76 ± 7.72 (1 missing)	− 6.47 ± 8.39 (1 missing)	0.399
pH	7.29 ± 0.13 (1 missing)	7.25 ± 0.16 (1 missing)	0.212
Comorbidities#			
Cardiovascular	26/35	23/31	0.993
Cancer	7/35	11/31	0.159
Metabolic	8/35	8/31	0.780
Pulmonary disease	4/35	4/31	0.855
Renal disease	9/35	13/31	0.163
Others	10/35	9/31	0.967

Causes of NOMI were identified and are presented in Table 3. The only significant differences were in the frequency of sepsis (*p* = 0.008) and pancreatitis (*p* = 0.016). More importantly, none of the diseases mentioned had a significant impact on survival.

**Table 3:** Causes of NOMI by treatment group

	Therapy		
	Interventional (number of patients)	Conservative (number of patients)	*p*
Cardiac emergencies	11	8	0.262
Postoperative condition	4	2	0.314
Sepsis	11	3	0.008
Hemorrhagic shock	4	3	0.856
Shock by other causes	1	3	0.367
Pancreatitis	–	6	0.016
Lung failure	–	4	0.098
Other diseases	–	6	–

Lactate serum levels, pH, base excess and high demand of noradrenaline before intervention were significantly associated with 30-day mortality adjusted for age and sex in the interventional group (Table 4).

**Table 4:** Association of biomarkers prior to intervention with 30-day mortality in the group with interventional treatment (*n* = 35)

Biomarker	Hazard ratio by 1 SD of the biomarker (95%–CI)*	p*	Threshold value	Youden index	Sens %	Spec %
Lactate; mmol/l	1.96 (1.26; 3.07)	0.003	> 7.68	0.49	65	83
pH	0.46 (0.28; 0.74)	0.001	< 7.32	0.49	65	83
Base excess	0.50 (0.28; 0.87)	0.015	< − 4.55	0.52	77	55
Noradrenaline; mg/kg/body weight	1.41 (1.02; 1.96)	0.040	> 0.0009	0.40	65	75

^*^Derived from Cox regression adjusted for age and sex

*SD* standard deviation; *CI* confidence interval; *Sens* sensitivity; and *Spec* specificity

Thresholds to discriminate between those who died and those who survived after 30 days were > 7.68 mmol/l for lactate, < 7.32 for pH, <  − 4.55 for base excess and > 0.0009 mg/kg/body weight for noradrenaline.

## Discussion

In our study, we assessed the impact of acute treatment of NOMI by local infusion of papaverine hydrochloride into splanchnic arteries in comparison with conservative noninterventional treatment. Our data indicate that intra-arterial therapy with papaverine hydrochloride increases the survival rate. Besides, an increased lactate serum level, a reduced pH, a negative base excess and a high demand for noradrenaline prior to intervention are associated with a poor patient outcome.

NOMI leads to bowel ischemia by a constriction of mesenteric vessels [15]. Therefore, intra-arterial administration of vasodilators may reduce vasospasm and thereby prevent mesenteric necrosis. For the treatment regimen and the applied vasodilators, studies published so far are based on small patient cohorts only [6, 16–25]. When considering papaverine for intra-arterial therapy, only four studies have been published until now with ten to 25 patients [6, 17, 19, 23]. No direct comparison with a conservative noninterventional treatment has been done so far. A study from 1977 showed that persistent mesenteric vasoconstriction could be disrupted by the selective administration of papaverine hydrochloride (60 mg/h continuous infusion dose) into the mesenteric artery [6] and nine of 15 patients survived (survival rate 60%). Clark et al. [17] achieved similar results (survival rate 45%, five out of 11 patients) with either papaverine hydrochloride (30–60 mg/h continuous infusion dose) or prostaglandin E2 (one patient, 0.6 to 1.5 mg/h infusion rate). Klotz et al. demonstrated a survival rate of 64% (nine out of 14 cases) in their interventionally treated cases using 53.5 ± 12.5 mg/h papaverine hydrochloride continuous infusion dose (bolus of 60 mg of papaverine in two cases) [19].

In comparison with previous studies, we gained a lower survival rate of 34.3% for patients treated with infusion therapy of papaverine hydrochloride in the setting of NOMI. The lower survival rate in our study may be explained by the fact that our study groups had higher lactate levels in comparison with previous research [19, 21, 25, 26]; lactate values on studies with local infusion of papaverine hydrochloride are only mentioned by Klotz et al. [19] (mean lactate for severe NOMI: 0.4 ± 0.5 mmol/l). While there was no linear relationship between lactate levels and the extent of ischemia in a study by Ambe et al. [27], higher levels of lactate were found in those with advanced ischemia. To compare survival with previous studies, we performed a threshold analysis on lactate regarding 30-day mortality. With an AUC of 0.79, lactate is suitable as a biomarker for differentiating between surviving and deceased patients [28]. Above the calculated cutoff lactate level of 7.68 mmol/l, 88% of these patients (15 of 17 patients) died. For interventionally treated patients with lactate levels below the threshold, survival rates were comparable to the studies on papaverine hydrochloride (averaged survival 52%) with a survival rate of 55% (ten of 18 patients), although our therapy regimen was different.

There has not been any recommendation or standardization so far regarding the dosage of papaverine hydrochloride for interventional treatment. Current research recommends a wide range of papaverine hydrochloride doses between 10 and 60 mg/h [6, 17, 19, 26, 29]. Thus, further research should suggest an optimal dosage for papaverine hydrochloride treatment of NOMI.

Furthermore, there are promising results using other vasodilators [18, 21, 24, 30], such as tolazoline, vasopressin, prostacyclins or prostaglandins. There has not been any recommendation thus far regarding the type of drugs applied for local arterial treatment. For example, in a recent experimental study by Mahlke et al. [30], iloprost, PGE1 and papaverine dilated pre-constricted human mesenteric arteries by a similar degree in vitro. Iloprost sensitivity was higher in vessels with a small lumen diameter, which is particularly suitable for the treatment for NOMI. Based on our experience, iloprost shows pronounced systemic side effects, like hypotension and tachycardia in clinical practice. Therefore, we prefer papaverine for intra-arterial therapy of NOMI.

In our study, we revealed increased lactate serum levels, a reduced pH, a negative base excess and a high demand for noradrenaline prior to intervention as factors leading to a poor patient outcome. In critical patient conditions, these laboratory parameters are generally associated with a worse outcome. Therefore, close observation of laboratory parameters, early screening for NOMI and well-timed intra-arterial therapy may increase the likelihood of survival.

Considering previous studies [6, 17–25] and our results, interventional treatment seems to have a distinctive advantage for patients’ outcome regardless of the scheme and vasodilator applied. To the best of our knowledge, this study is the first to directly compare an interventional treatment with papaverine hydrochloride and conservative treatment approach of NOMI. Besides, in our study cohort, size of patients was by far the largest to date.

The study has several limitations. First, the study design was retrospective. Although the results are promising in terms of mortality reduction, there are no prospective, randomized studies that can prove the benefits of intra-arterial vasodilator therapy.

Another limitation lies in the comparability of both groups. As serum lactate levels at the time of the diagnosis differed substantially (8.8 ± 6.8 mmol/l vs. 12.7 ± 7.9 mmol/l, *p* = 0.025), survival analysis was adjusted for covariate lactate. There was still a distinct survival benefit of interventional therapy in patients with NOMI (*p* = 0.005). Further, we used the SOFA score, which is a consensus established by the European Society for Intensive Care Medicine (ESICM) for the objective description of organ dysfunction, to compare subgroups regarding expected mortality. Both groups had similar SOFA scores (10.94 ± 2.5 vs. 10.93 ± 2.9, *p* = 0.993) at the time of diagnosis. Furthermore, there were no other significant differences according to demographic and laboratory data. Finally, we cannot exclude that there are further relevant differences (e.g., differences in medical care and protocols for early detection of sepsis and SIRS) between both groups that could have influenced our results.

In conclusion, our data suggest a significant benefit of local intra-arterial papaverine infusion therapy regarding 30-day mortality in the treatment for NOMI in comparison with a conservative therapy approach. To improve the probability of survival, in the first step, diagnostics of NOMI should be much more sensitive to detect patients at an earlier stage of ischemia. In the second step, standard operation procedures at both centers should include interventional therapy, which requires appropriate training as well.

High lactate levels, low pH and high base excess and high demand for catecholamines are associated with a poor patient outcome of interventional treatment. If NOMI is suspected either by imaging or clinical signs, we would recommend an immediate selective angiography to confirm diagnosis, followed by an intra-arterial papaverine infusion as treatment for NOMI.
